# Graphene-based carbon-layered electrode array technology for neural imaging and optogenetic applications

**DOI:** 10.1038/ncomms6258

**Published:** 2014-10-20

**Authors:** Dong-Wook Park, Amelia A. Schendel, Solomon Mikael, Sarah K. Brodnick, Thomas J. Richner, Jared P. Ness, Mohammed R. Hayat, Farid Atry, Seth T. Frye, Ramin Pashaie, Sanitta Thongpang, Zhenqiang Ma, Justin C. Williams

**Affiliations:** 1 Department of Electrical and Computer Engineering, University of Wisconsin—Madison, Madison, Wisconsin 53706, USA; 2 Materials Science Program, University of Wisconsin—Madison, Madison, Wisconsin 53706, USA; 3 Department of Biomedical Engineering, University of Wisconsin—Madison, Madison, Wisconsin 53706, USA; 4 Department of Electrical Engineering and Computer Science, University of Wisconsin—Milwaukee, Milwaukee, Wisconsin 53211, USA; 5 Department of Biomedical Engineering, Mahidol University, Bangkok 73170, Thailand

## Abstract

Neural micro-electrode arrays that are transparent over a broad wavelength spectrum from ultraviolet to infrared could allow for simultaneous electrophysiology and optical imaging, as well as optogenetic modulation of the underlying brain tissue. The long-term biocompatibility and reliability of neural micro-electrodes also require their mechanical flexibility and compliance with soft tissues. Here we present a graphene-based, carbon-layered electrode array (CLEAR) device, which can be implanted on the brain surface in rodents for high-resolution neurophysiological recording. We characterize optical transparency of the device at >90% transmission over the ultraviolet to infrared spectrum and demonstrate its utility through optical interface experiments that use this broad spectrum transparency. These include optogenetic activation of focal cortical areas directly beneath electrodes, *in vivo* imaging of the cortical vasculature via fluorescence microscopy and 3D optical coherence tomography. This study demonstrates an array of interfacing abilities of the CLEAR device and its utility for neural applications.

## Abstract (web summary)

Monitoring neuronal activity in the rodent *in vivo* brain is commonly done using micro-electrode arrays but these devices are not normally compatible with optical technologies. Here the authors design a transparent and flexible electrode array based on graphene that allows them to combine electrophysiological recordings with optogenetic and imaging experiments.

## Graphical abstract

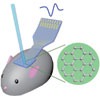

Neural interfaces enable a connection between nervous tissue and the *ex vivo* environment. These devices are not only useful for neuroscience research but also provide therapy for patients afflicted with a multitude of neuronal disorders, such as Parkinsons disease, spinal cord injury and stroke. The advent of optogenetics, a new technique involving genetic modification of neural cells to make them susceptible to light stimulation, has not only revolutionized neuroscience research, but also transformed the requirements for neural interfacing devices^1^. It is now desired to optogenetically stimulate the cortex with light while simultaneously recording the evoked response. Neural surface electrode arrays, such as micro-electrocorticography (micro-ECoG) devices, strike a balance between invasiveness and recorded signal quality^2^,^3^,^4^,^5^. However, these devices use opaque metallic conductive materials. Thus, it is possible to stimulate around the electrode sites, but not directly at the electrode–tissue interface^6^. Additional advancements in *in vivo* imaging modalities could provide valuable information regarding the tissue response to implanted electrode arrays, and help correlate tissue behaviour with recorded signals^7^. To date, these methods have mainly been used to image tissue surrounding micro-ECoG electrode sites, as imaging at the electrode–tissue interface is infeasible, due to the conductor opacity^7^. Therefore, correlation of imaging data with neural signals has been difficult. Development of completely transparent micro-ECoG devices would be valuable for the advancement of neural research in terms of both optogenetics and cortical imaging applications, and could lend insight into brain function, further improving therapeutic neural interface application.

Previously, transparent micro-ECoG arrays have been fabricated using indium-tin oxide (ITO), a transparent conductor commonly used in solar cells^8^,^9^. ITO, however, is not ideal for employment with micro-ECoG technology, for a variety of reasons. First, ITO is brittle and thus not conducive to use in flexible electronics applications^10^. As a large benefit of micro-ECoG is its ability to conform to the cortical surface, the brittleness of the ITO is limiting. Second, ITO deposition requires high-temperature processing not suitable for use with the low glass transition temperature Parylene substrate of the micro-ECoG array^11^,^12^. Third, ITO has process-dependent transparency, which is rather limited in the ultraviolet and infrared^13^,^14^. Neural imaging and optogenetics applications require the use of a wide range of wavelengths (from ultraviolet to infrared) for stimulating various opsin types and visualizing fluorescently tagged cells. Therefore, for maximum versatility, neural interfaces that can allow light transmission with high transparency over a broad spectrum are beneficial.

Owing to the drawbacks of ITO, transparent micro-ECoG technology has yet to be demonstrated for chronic implementation. Towards the creation of a completely transparent, chronically stable device useful over a broad light spectrum, we propose a graphene-based transparent micro-ECoG array. Graphene has been widely researched for a variety of applications due to its excellent electrical/thermal conductivity, transferability, strength and tunable electronic properties^15^,^16^,^17^. Furthermore, the biocompatibility and broad-spectrum transparency, flexibility and mass-producibility of graphene make it an ideal candidate for replacement of ITO in neural interfacing devices^18^,^19^,^20^,^21^. Here we report a fabrication method for a graphene-based carbon-layered electrode array (CLEAR) device for neural interfacing and demonstrate its long-term *in vivo* stability and viability for electrophysiology, optogenetics and cortical imaging experiments.

## Results

### CLEAR device fabrication

**Figure 1:**
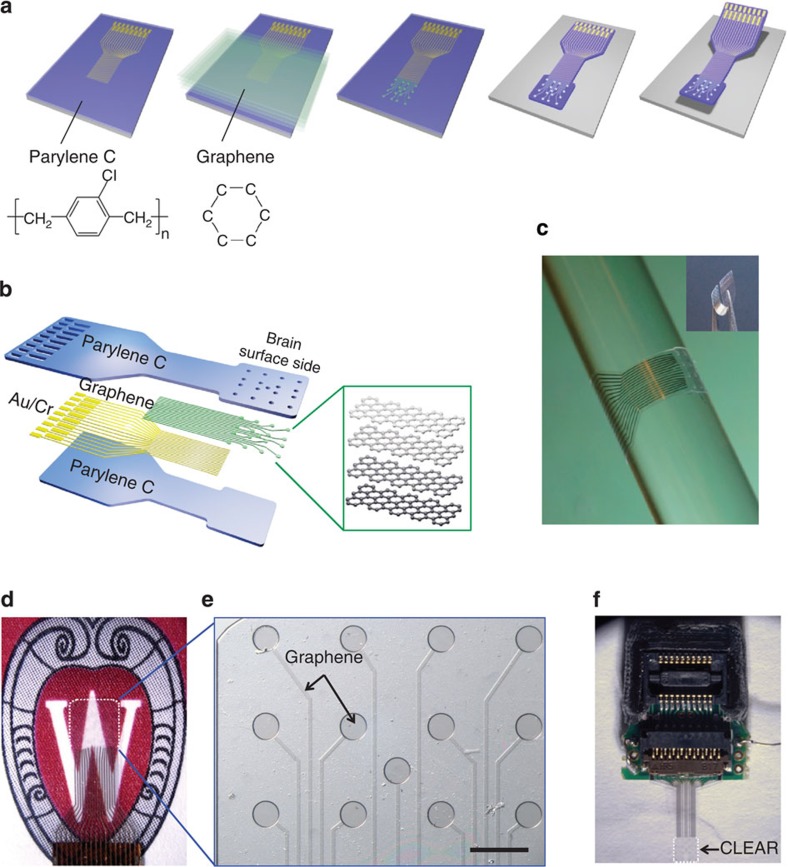
CLEAR micro-ECoG device. (**a**) Basic fabrication process: metal patterning of traces and connection pads on Parylene C/silicon wafer. The silicon wafer is the handling substrate. Transfer and stack four monolayers of graphene sequentially. Graphene patterning to form electrode sites. Second Parylene C deposition and patterning to form device outline. Removal of device from silicon wafer. (**b**) Diagram of CLEAR device construction showing the layered structures. (**c**) Demonstration of CLEAR device flexibility. The device is wrapped around a glass bar with a radius of 2.9 mm. (**d**) Rat-brain-sized CLEAR device: outlined by white dashed line (electrode area of 3.1 × 3.1 mm^2^). (**e**) Close-up of rat-sized device showing transparent graphene electrode sites and traces on a Parylene C substrate. This side touches brain surface. Scale bar, 500 μm. (**f**) Mouse-brain-sized CLEAR device with zero insertion force (ZIF) PCB connector (electrode area of 1.9 × 1.9 mm^2^).

Following graphene characterizations using Raman spectroscopy (as described in the Supplementary Information), implantable graphene neural electrode arrays were fabricated on a 4-inch silicon wafer. Figure 1a shows a simplified schematic of the fabrication process. Briefly, wafers were coated with Parylene C films using a chemical vapour deposition process. Next, the connection pads and initial portions of the traces were patterned with gold via electron beam evaporation and lift-off techniques. The use of gold for the traces and pads was necessary to ensure a good mechanical connection to the zero insertion force printed circuit board (PCB) connectors used for reading the brain signals into the computer. The electrode sites and portions of the traces that were to be in contact with the brain were left for the subsequent graphene transfer and patterning, such that the brain contact area of the electrode would be transparent. Following metallization, four graphene monolayers were transferred and stacked sequentially onto the wafer surface, using the wet transfer technique described in Supplementary Figs S1–S4. A sacrificial layer of SiO_2_ was then deposited to protect the graphene layers from being etched in the reactive ion etching (RIE) steps to follow. After that, the graphene was patterned to form the electrode sites using RIE and another insulating layer of Parylene C was deposited. RIE was then employed to expose the electrode sites and pads, and form the array outlines. Finally, the devices were peeled from the wafer, the SiO_2_ protection layer was removed by wet etching and the arrays were inserted into the PCB connectors. The detailed fabrication process and the graphene characterization using Raman spectroscopy are described in Supplementary Figs S5 and S6, respectively.

### Device characterization

**Figure 2:**
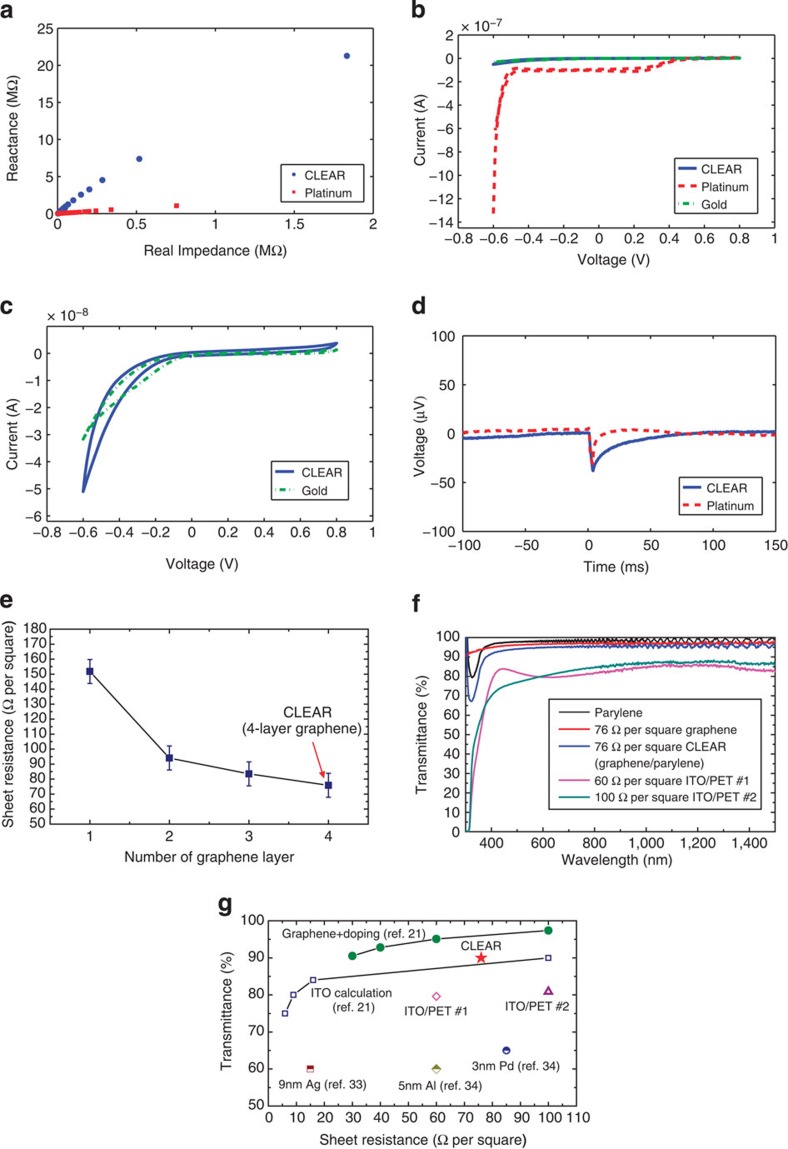
Electrode characterizations. (**a**) Electrical impedance spectra for CLEAR and platinum micro-ECoG devices in saline. The *x* axis represents real impedance and *y* axis represents imaginary impedance. Each point was taken at a different frequency, between 10 Hz and 31 kHz. (**b**) Average CV results over 16 electrode sites on CLEAR, gold and platinum micro-ECoG arrays. (**c**) Average CV results for 16 electrode sites on CLEAR and gold micro-ECoG arrays. (**d**) Average artefact effect test results for CLEAR and platinum micro-ECoG devices, with light applied to a single electrode site on each device via an optical fibre attached to a blue laser, with an application of 63.7 mW mm^−2^ power for 3 ms. (**e**) Trend of sheet resistance as a function of the number of graphene layer. The error bar represents the s.d. of sheet resistance extracted from five sample measurement. (**f**) Light transmittance test results for 76 Ω per square four graphene monolayers on a 15-μm Parylene C film (CLEAR), 76 Ω per square four graphene monolayers only (Graphene), Parylene C film only (Parylene), 60 Ω per square ITO/PET and 100 76 Ω per square ITO/PET film. (**g**) Transmittance versus sheet resistance graph for various conducting materials (graphene, ITO, ultrathin metals).

The patency of the completed devices was verified via electrical impedance spectroscopy. Impedance spectra were obtained for each electrode site using an Autolab PGSTAT12 potentiostat. Devices were connected to the Autolab machine via the PCB connectors, which were connected to a passive 32-channel Tucker-Davis Technologies (TDT) head stage. Impedances were evaluated at 30 different frequencies, ranging from 10 to 30,937 Hz. If electrode sites had impedance values <600 kΩ at 1 kHz frequency, they were considered to be viable for implantation. One kilohertz frequency was selected for evaluation, because it is known to be a common benchmark for neural impedance analysis^22^,^23^. Figure 2a shows representative electrical impedance spectra for CLEAR and traditional platinum micro-ECoG devices tested in saline. It is apparent from the plot that the phase angle is higher in the case of the CLEAR device. This means that the value of the reactance is higher for the graphene sites than for the platinum. However, the average magnitude of the impedance at 1 kHz frequency was only slightly higher for the CLEAR device than for the platinum array (243.5±5.9 kΩ for graphene versus 188.8±92.9 kΩ for platinum). As the signal-recording apparatus involves the use of a high-input impedance amplifier, the reactance difference is not expected to have adverse effects on the signal recordings.

In addition to impedance spectroscopy, cyclic voltammetry (CV) was performed on select CLEAR devices and on devices with gold and platinum electrode sites for comparison purposes. CV was also performed using the Autolab system. CV scans were taken from −0.6 to 0.8 V with a step potential of 0.0105 V and a scan rate of 0.0500 V s^−1^ (refs 24, 25). The voltage range was chosen so as to stay within the water window. Average CV curves for gold, platinum and CLEAR devices are shown in Fig. 2b,c. The CV results for the platinum device were drastically different than those for either the gold or the CLEAR devices. Although this demonstrates that the platinum electrode sites have a greater charge-carrying capacity, it does not rule out the use of CLEAR devices for neural interfacing applications. In fact, the average CV curves for the CLEAR and gold electrode sites were similar. As gold has proven itself as a reliable material for recording electrode sites^26^,^27^, this suggests that the graphene conductors will also be sufficient.

The construction of completely transparent neural surface electrode arrays enables unique research capabilities in combining optogenetics^28^ with electrophysiology, by allowing for delivery of light stimuli through the array, directly to the brain region from which the recordings are obtained. Even though the electrodes are optically transparent, an artefact is still present when high-intensity light is applied directly onto an electrode site. The artefact is generally smaller in amplitude and temporally different than the optogenetically evoked neural signal. The artefact is reproducible and can be characterized by testing the electrode site in saline, implanting into wild-type animals or testing in cadaver experiments. To test the artefact, the devices were placed face down in saline solution and a 200-μm optical fibre connected to a 100-mW, 473-nm diode laser was used to shine light onto the backs of the electrode sites. The light pulses were delivered by applying 3 V to the laser for 3 ms (up to 63.7 mW mm^−2^). Figure 2d shows the electrical pulses elicited by the light impingent on graphene and platinum sites. From the plot, it appears that the amplitude of the artefact is similar for both the graphene and the platinum sites, although the platinum electrode returns to baseline more quickly. We expect the main mechanism of the artefact is similar to when light is applied to a conventional metal electrode, which is known as Becquerel effect, a classical photoelectrochemical effect first demonstrated by Becquerel in 1839 (refs 29, 30). More details are discussed in the optogenetic testing section with further *in vivo* experiments and analysis.

Another important characteristic for optogenetic application of the CLEAR device is the amount of light transmitted through the graphene electrode sites and Parylene substrate. As the intention for the transparent device is that one can both image and project light onto the underlying brain tissue, it is important that a large percentage of the light impinging on the array is transmitted through the device. In a material, inherent tradeoffs exist between transparency and conductivity. In general, by increasing the thickness of a material, the conductivity is increased but the transparency is decreased. This tradeoff also exists in the graphene case. Bae *et al.*^21^ reported an increase in conductance (that is, sheet resistance decrease) and a decrease in transmittance when going from one to four graphene monolayers. To verify this trend in our chemical vapor deposition (CVD) graphene, sheet resistance was extracted using the transmission line method (Supplementary Fig. S7). Figure 2e shows the sheet resistance decrease from 152 to 76 Ω per square for one-layer and four-layer graphene. This shows a similar sheet resistance trend to the previous report. Note that relatively higher values than the reported values are attributed to their HNO_3_-doped graphene^21^. For the CLEAR device, four-layer graphene was chosen to have the minimum sheet resistance while maintaining around 90% transmittance. In addition, the four-layer graphene is expected to have better yield than fewer-layer graphene by compensating possible mechanical tear-off of each graphene layer during the transfer process.

A plot of the light transmittance versus wavelength for Parylene, four-layer graphene, CLEAR (four-layer graphene/parylene) and ITO/PET (polyethylene terephthalate) films is shown in Fig. 2f. The measurements were taken for light wavelengths from 300 to 1,500 nm using a ultraviolet–visible spectrometer. The sinusoidal shape of the transmittance curve is common for the Parylene C material^31^. For the CLEAR device, an average of ~90% of the light impinging on the substrate is transmitted at the desired wavelengths (470 nm for excitation of channelrhodopsin and 570 nm for halorhodopsin). This is similar to previously reported results and is sufficient for numerous optogenetic and imaging applications^9^,^32^. To compare the CLEAR and ITO films, commercial ITO/PET films with similar sheet resistance values (60 and 100 Ω per square) were measured using the same instrument. For both films, an average of around 80% transmittance in the visible-infrared range and dramatically reduced transmittance near the ultraviolet range are shown. In addition, a transmittance difference of ~10% is shown in the 420–440 nm range, which is attributed to the different properties of the ITO films.

For a more quantitative comparison between the graphene, ITO and ultrathin metal films, the measured transmittance and sheet resistance values are plotted in Fig. 2g, along with various values reported in the literature. Sheet resistance is widely used to evaluate and compare conducting materials^13^,^14^,^21^,^33^,^34^. From the plot we can see that for the given sheet resistance range, graphene films show >90% transmittance while ITO and ITO/PET films show closer to 80% transmittance. Moreover, the ultra-thin metals show much less transmittance (~60%). This comparison indicates that the light transmittance through the CLEAR devices is superior to ITO and ultra-thin metals.

### *In vivo* validation of neural signal and impedance recordings

To demonstrate the *in vivo* performance of the CLEAR devices, the arrays were implanted in four rats and five mice, one wild-type mouse for imaging and four Thy1::ChR2 mice for imaging and optogenetic testing. Table 1 describes the type of implantation performed in each case and the type of data collected from each animal. Three of the rat implantations were bilateral, with CLEAR and the platinum devices implanted within the same animal for a fair comparison and to reduce overall animal numbers.

**Figure 3:**
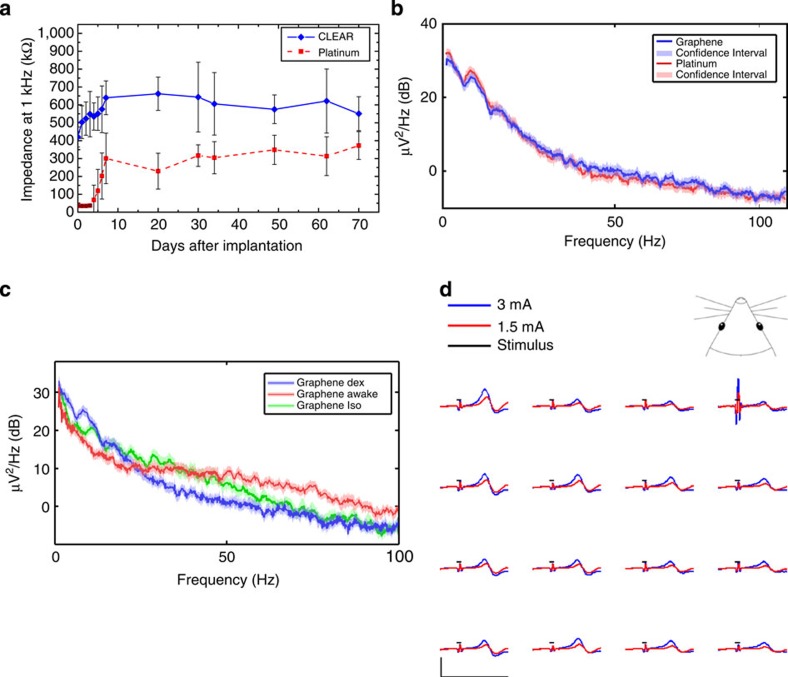
*In vivo*-recorded signal characterizations. (**a**) Average longitudinal 1 kHz impedance values for CLEAR and platinum micro-ECoG devices implanted in the same animal. The error bar represents s.d. of impedance extracted from 16-channel measurement. (**b**) Baseline signal power spectra for the CLEAR and platinum devices under dexmedetomidine with 95% confidence interval using jack knife resampling. (**c**) Baseline signal power spectra for a CLEAR device under two analgesic conditions, dexmedetomidine and isoflurane, compared with an awake condition. (**d**) Sensory evoked potentials recorded by the CLEAR device via electrical stimulation of the sciatic nerve on the hind leg of the rat, contralateral to the array. The device was implanted over somatosensory cortex. Stimuli were applied for 1 ms at 3 and 1.5 mA current levels. The *x* scale bar, 50 ms; *y* scale bar, 100 μV.

Average electrode site impedance (measured from the first day of device implantation) for CLEAR and platinum micro-ECoG arrays implanted in the same animal are plotted over time in Fig. 3a. Both devices experienced a steep rise in impedance within the first 10 days after implantation, most probably due to the initial tissue response to the implanted arrays^22^. After this initial sharp increase, the impedances appear to have plateaued, with some minor day-to-day fluctuations. The shape of these impedance curves is characteristic of epidurally implanted micro-ECoG devices^7^. There was no statistically significant difference between the impedance changes for the CLEAR and platinum micro-ECoG devices over the entire implantation period, suggesting that the CLEAR device will perform, as well as the platinum device over the explored time period.

Figure 3b shows power spectra for the baseline local field potentials recorded by single channels on the CLEAR and platinum micro-ECoG devices in the 1–100 Hz frequency range. As with the longitudinal impedance data, there is little difference between the signals recorded by the two different arrays. The 95% confidence intervals for the CLEAR and platinum devices overlap, suggesting little difference between the signals recorded by the different devices. This similarity is shown in the high frequency range (1–600 Hz) as well (Supplementary Fig. S8a). In addition, Fig. 3c shows the baseline spectra obtained with the animals anaesthetized using two different anaesthetics, dexmedetomidine and isoflurane. Dexmedetomidine induces sleep-like rhythms, with more low frequency oscillations, while isoflurane suppresses general signal power, as shown in the figure. Both dexmedetomidine and isoflurane suppress high gamma −6 dB at 80 Hz, a 75% reduction in amplitude compared with the awake condition. This suggests that clear electrodes are able to pick up the high-gamma signals that have been found to be useful for ECoG-based brain–computer interface applications^35^,^36^.

In addition to baseline signal recordings and impedance spectroscopy measurements, rats were tested for electrical-evoked potentials. In these experiments, the hindlimbs of the animals were stimulated with surface electrodes placed above and below the sciatic nerve. Stimuli consisted of 1 ms biphasic electrical pulses, with amplitudes varying from 1 to 3 mA. Evoked potentials were recorded with stimuli applied both ipsilateral and contralateral to the implanted devices, to verify that the result was, in fact, the somatosensory response to the electrical stimulus. If this was true, evoked potentials would be seen only when the stimuli were applied contralateral to the implanted electrode array, due to the crossing of the neural pathways in the brainstem and spinal cord. Figure 3d shows a summary of the evoked potential results for the contralateral implanted CLEAR device at two stimulation levels. The results show a neural response with ~100 μV evoked potentials. Signals recorded in the device with ipsilateral leg stimulation lacked the neural evoked potential that was found on the contralateral side (Supplementary Fig. S8b). In general, the results displayed in Fig. 3 show that the graphene electrode sites are capable of recording both spontaneous baseline activity and evoked neural signals with the same level of clarity as the platinum sites, and generally similar impedance behaviour and stability over time.

### Optogenetic testing

**Figure 4:**
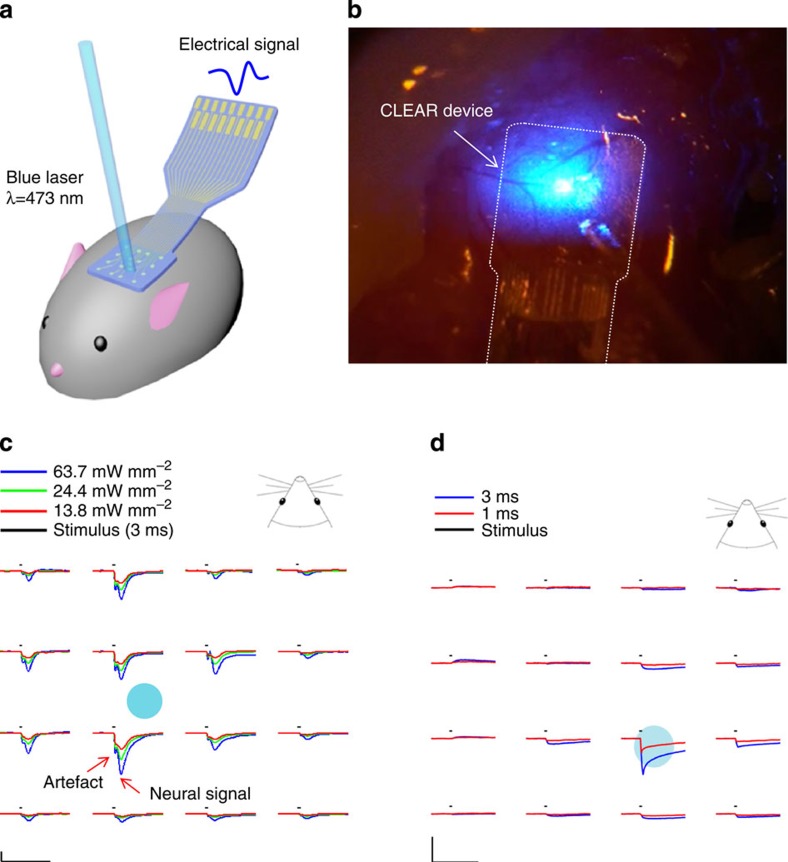
Optogenetic experimentation through the transparent CLEAR device. (**a**) Schematic drawing of opto-experimental setup, showing the CLEAR device implanted on the cerebral cortex of a mouse, with an optical fibre delivering blue light stimuli to the neural cells. (**b**) Image of a blue light stimulus being delivered via an optical fibre, through the CLEAR device implanted on the cortex of a Thy1::ChR2 mouse. (**c**) Optical evoked potentials recorded by the CLEAR device. X-scale bars represent 50 ms, y-scale bars represent 100 μV. (**d**) Post-mortem control data with the laser set at 24.4 mW mm^−2^ with the light impingent on electrode site 11 of the CLEAR device. The *x* scale bar, 50 ms; *y* scale bars, 100 μV.

Three Thy1::ChR2 mice were terminally implanted with a CLEAR device for the purpose of optogenetic evaluation. These mice had neurons expressing the channelrhodopsin-2 protein, making them susceptible to excitation when exposed to blue (473 nm) light. The average evoked response is shown for three different light-intensity stimulation levels in Fig. 4c. The stimulus time was 3 ms. The initial peak corresponds to the expected stimulus artefact and the second, longer peak is the evoked neural response. The stimulus artefact peak is detected ~3 ms after the stimulus onset, while the evoked neural response peak has latency ~7.7 ms following the stimulus onset. These latencies are highly dependent on the details of the eventual intended experimental requirements (that is, light intensity, stimulus duration and interstimulus interval). This leads to the logical consequence that in many experimental paradigms the stimulus artefact will be completely distinguishable from the evoked neural response, and in others there may be considerable overlap.

Once experimentation was complete, the animals were killed and a control experiment was conducted with the CLEAR micro-ECoG array placed on the somatosensory cortex of the killed animal to verify that the signals recorded were from neurons affected by the light stimulation and not due to the artefact. From Fig. 4d we can see that the signals obtained from the post-mortem control experiment are different from the signals recorded from the living animal, both temporally and according to amplitude of the negative peaks. These results demonstrate that the signals in Fig. 4c were evoked neural responses to the light stimulation.

An experiment to demonstrate the increased spatial resolution of the CLEAR device was performed using a lower-power light intensity of 1.24 mW mm^−2^. As this lower-power light results in less spatial spread, the volume of neural tissue activated is also more confined and its response could be detected in a more focal area. Supplementary Fig. S9a shows that with low-intensity light stimulation it is possible to activate a relatively focal brain region (predominantly on one electrode site in this case). In this case, the amplitude of neural response was also reduced compared with the higher light intensity used in Supplementary Fig. S9b (24.4 mW mm^−2^). This is attributed to the reduced volume of stimulated neurons caused by the reduced radius and depth of the light stimulation^37^.

For more detailed artefact analysis, we investigated the dependency of the light stimulation timing and power. Optical stimulus artefact is an important consideration for any kind of recording electrode and is difficult to avoid due to the ionic charge transfer layer at the conductor–electrolyte interface. Electrically conductive materials in ionic solution are in general subject to the Becquerel effect, a classical photoelectrochemical effect first demonstrated by Becquerel in 1839 and known as the main mechanism of the stimulus artefact for conventional metal electrodes^29^,^30^. Graphene itself has metal-like zero band gap nature and it is a conducting material without further band gap engineering^15^,^17^. Therefore, the classical photoelectric effect is unlikely to contribute to the artefact, as graphene has a relatively high work function (4.5 eV). According to the experiments in this study, the amplitude level of the artefact was similar in both graphene and platinum electrodes (Fig. 2d), suggesting a similar underlying mechanism. Additional experiments also showed similar dependency on the light stimulation time and power as to the metal electrode case (Supplementary Fig. S10) ( http://www.openoptogenetics.org/index.php?title=Light-Induced_Artifact.)^38^. The amplitude of the artefact increased as the stimulation time was increased from 3 to 25 ms, which is similar to what has been reported previously in varying metal electrodes ( http://www.openoptogenetics.org/index.php?title=Light-Induced_Artifact.). The peak of the artefact also increased temporally as stimulation duration increased. In addition, the light power dependency was similar to that of the metal electrode^38^, where the artefact decreased with decreasing stimulation power (Supplementary Fig. S10b). It is interesting to note that the artefact is significantly lowered when the stimulus power are reduced, which is expected from traditional metal electrode behaviour. It is therefore important to note that the stimulus artefact may be reduced or eliminated with lower-level light stimulus, such as would be subjected during traditional imaging paradigms. Other strategies can also be employed to further characterize and reduce the stimulus artefact, as have been proposed in other optogenetic studies^39^, and ones that have been similarly successful in dealing with electrical stimulation artefacts^40^. Further study will be necessary to fully characterize the overall performance of the graphene devices and source of potential artefacts.

### *In vivo* imaging

**Figure 5:**
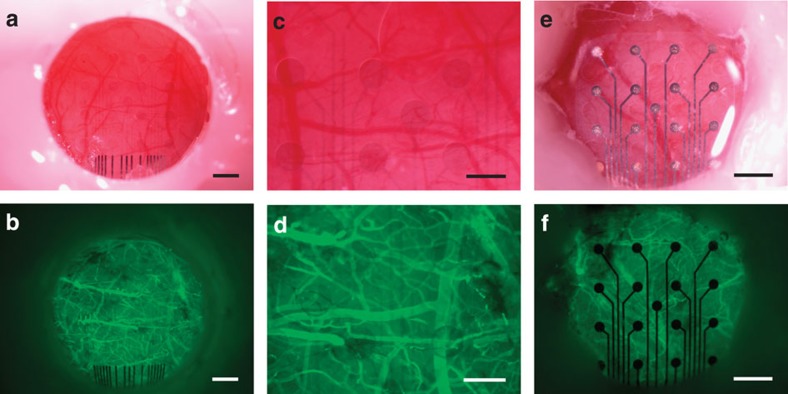
Representative *in vivo* images of the cortical vasculature seen through CLEAR device. (**a**) Bright-field image of CLEAR device implanted on the cerebral cortex of a mouse beneath a cranial window. (**b**) Fluorescence image of same device shown in **a**. Mouse was given an intravenous injection of fluorescein isothiocyanate–dextran to fluorescently label the vasculature. (**c**,**d**) Higher magnification bright-field and fluorescence images of same device shown in **a** and **b**, respectively. (**e**,**f**) Bright-field and fluorescence images of standard rat-sized micro-ECoG arrays with platinum electrode sites, respectively. Scale bars, 500 μm (**a**,**b**), 250 μm (**c**,**d**), 750 μm (**e**,**f**). *In vivo* vasculature imaging was repeated in three rats, each with a CLEAR and platinum microECoG array. Images were representative of presented and previously published data^7^,^50^.

A subset of the implanted animals was imaged via the cranial window imaging method previously described by Schendel *et al.*^7^ Representative images of the cortical vasculature through the CLEAR micro-ECoG device are shown in Fig. 5a–d. Images in the left column were taken in bright field, while those on the right were taken under blue (470 nm) light with the aid of a tail vein injection of fluorescein isothiocyanate–dextran to fluorescently label the vasculature. These images demonstrate the clarity of the graphene electrode sites and the ability to view the underlying cortex and cerebral vasculature through the CLEAR device. Blood flow movie recordings taken through the transparent graphene electrode sites can be seen in Supplementary Movies 1 and 2. Figure 5e,f show cranial window images of a platinum micro-ECoG array, with the electrode sites and traces clearly visible.

**Figure 6:**
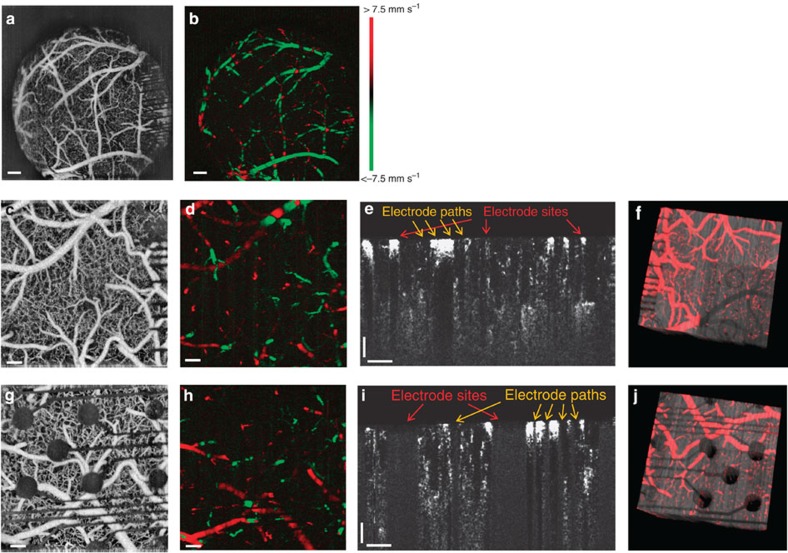
Optical coherence tomography through CLEAR and platinum devices. (**a**,**c**) Maximum intensity projection (MIP) of OCT angiogram showing cortical vasculature visible through the CLEAR micro-ECoG device (FOV 2.8 × 2.8 mm^2^ and 1.1 × 1.1 mm^2^, respectively). (**b**,**d**) Doppler blood flow velocity image showing the directionality of blood flowing through the vasculature below the CLEAR device (FOV 2.8 × 2.8 mm^2^ and 1.1 × 1.1 mm^2^). Red colour represents blood flowing towards the lens and green colour represents blood flowing away. (**e**) Cross-sectional angiogram after contrast enhancement and de-shadowing (CLEAR device). (**f**) 3D visualization of the vasculature (red colour) overlaid on the structural data (grey) (CLEAR device). (**g**) MIP of angiogram through a platinum micro-ECoG device. (**h**) Corresponding Doppler blood flow velocity measurements. (**i**) Corresponding cross-sectional angiogram. (**j**) Corresponding 3D visualization of vessel structure for a micro-ECoG array with platinum electrode sites. Scale bars, 200 μm (**a**,**b**), 100 μm (**c**–**i**).

In addition to fluorescence imaging of the cortical vasculature, optical coherence tomography (OCT) imaging demonstrates the ability of the CLEAR device based on its high transparency in the infrared spectral range. The structure of the cerebral vasculature can be captured as a three-dimensional (3D) OCT angiogram^41^,^42^,^43^ through the device, as shown in Fig. 6a,c. Furthermore, two typical velocity profiles^44^,^45^,^46^,^47^ of blood flow below the CLEAR device are demonstrated in Fig. 6b,d. In these images, dark lines produced by the gold traces are visible on the right side; however, the centre of the array is clear with no dark lines and vessels are easily visible. Figure 6e shows a typical cross-sectional angiogram after contrast enhancement under the CLEAR device. The OCT system is able to detect the vessels under electrode sites and electrode traces. The 3D visualization^48^ of the angiography data is presented in Fig. 6f. The structural data is shown in grey and vessels are shown in red. Similar data for the platinum device is shown in Fig. 6g–j, indicating the opaqueness of the platinum electrode sites. The 3D structural movies for CLEAR and the platinum devices can be seen in Supplementary Movies 3 and 4, respectively. The comparison shows the advantage of the CLEAR device over the conventional platinum device. Moreover, it is important to note that OCT uses infrared light wavelengths, which may create artefacts with ITO-based devices, depending on the fabrication process.

## Discussion

The results of this study demonstrate that the CLEAR micro-ECoG device is capable of recording neural signals with the same degree of clarity as the platinum array, and have a comparable longitudinal tissue response. Unlike the platinum array, the CLEAR device allows for optogenetic stimulation and both fluorescence and OCT imaging directly through the electrode sites, due to the broad spectrum transparency of graphene. Increasing the density of the electrode sites on the CLEAR device would allow for increased spatial resolution of the recorded signals. If the electrode site density were to be increased using an opaque metal electrode array, the increased amount of metal material, not only in the electrodes but also the traces, would block an ever increasing proportion of the stimulation light. There is probably a fundamental limitation to the spatial resolution of cortical surface recordings, which is still an active area of investigation in the field^4^. There is however a complementary push in the field to increase the overall channel count of surface electrodes^4^, which leads to a similar issue in the opacity of not only the electrode sites, but more importantly the corresponding increased density of the electrode lead lines. In the majority of electrode layout designs, the lead lines traverse through the spaces between the more interior electrodes, which for increasing channel counts would result in an increasingly higher density of opaque lead lines in the centre of the device, if traditional metallic conductors were used. Although future studies will be necessary to determine the long-term stability of this device, both in terms of biocompatibility and recorded signal quality, these findings and previous studies reporting the biocompatibility of CVD graphene suggest that the CLEAR device is a viable micro-electrode array for neural interfacing applications. This graphene device is superior to the present ITO-based transparent electrode technology for its dramatically increased mechanical flexibility^21^ and greatly enhanced transparency in the relevant spectral ranges. The tunable electrical properties of graphene could lead to future integration of active electronic elements into these devices. Future directions for transparent neural interfacing studies may include exploration and implementation of these properties with CLEAR technology.

Although the current implementation of this technology is centred around high-density surface arrays, the fabrication techniques described in this manuscript are readily amenable to making penetrating multi-electrode arrays, which would be capable of recording single unit, multi-unit and local fields. The only major obstacle to this potential line of work is the challenge involved in inserting these ultra-flexible devices. There have been a number of studies that have addressed the issue of inserting flexible polymer-based penetrating devices^49^, and these same strategies would be viable with the CLEAR technology. In addition, one of the proposed advantages of graphene is also to be able to integrate graphene-based transistors in a monolithic manner to integrate active circuitry into the device. The addition of circuitry will further necessitate the transparency of the electrodes, traces and circuit elements.

The advantages of the CLEAR technology are especially pronounced in optical imaging applications. As demonstrated in the present OCT experiments, the CLEAR electrodes present dramatic improvements in 3D imaging applications over conventional metal electrodes. Particularly in advanced imaging modalities such as multiphoton confocal imaging and OCT, the superior performance of graphene in the infrared spectrum makes it an attractive choice over other transparent materials such as ITO. There are also emerging techniques using multiphoton excitation and holography that would allow both finer resolution of stimulation and the ability to stimulate at depth within the cortex, both of which would benefit from the wide spectrum transparency of the graphene electrode material. It is also worth noting that the optical stimuli presented in this study are at the upper range of optogenetic inputs, and thus represent the worse case scenario for this technology. The proposed imaging applications use significantly lower power levels, with much reduced temporal activation patterns. For all intensive purpose, the graphene electrodes could be considered to be ‘artefact free’ for the majority of their intended imaging applications.

Optogenetic experiments, *in vivo* imaging of the cortical vasculature via fluorescence microscopy and OCT reveal additional unique abilities of these devices, made possible by their broad spectrum transparency. This study demonstrates the wide array of interfacing abilities of the CLEAR device and indicates its broad utility for neural and other biomedical applications.

## Methods

### Device fabrication

Silicon wafers were coated with 15 μm of Parylene C using a CVD process (SCS Labcoter 2 Parylene Deposition System). Ten nanometres of chromium (Cr) and 200 nm of gold (Au) were evaporated onto the Parylene substrate and patterned using lift-off techniques to form connection pads and the initial portions of the electrode traces.

After metal deposition, four monolayers of graphene were transferred onto the substrate following the procedure described in the supplement. The stacked graphene was then coated with a 30-nm SiO_2_ sacrificial layer, to protect against damage during subsequent RIE steps. Next, the graphene and SiO_2_ layers were patterned via RIE with oxygen plasma, to create 16 electrode sites connected to each of the gold pads.

Subsequently, another 10 μm of Parylene C were deposited via CVD and patterned by photolithography and RIE, to create the array outlines and to open the electrode sites and pads. The arrays were released from the silicon wafer via immersion in de-ionized water. Finally, the protective SiO_2_ layer was removed using 1:6 buffered oxide etchant. A polyimide stiffener was then bonded to the pad region of each device to increase the thickness of the array enough to ensure a good connection to the zero insertion force PCB (Imagineering Inc., Elk Grove Village, Illinois). The detailed process diagram is provided in Supplementary Fig. S5.

### Surgical implantation

All animal procedures were approved by the Institutional Animal Care and Use Committee at the University of Wisconsin–Madison. Surgical procedures and *in vivo* imaging sessions were performed under anaesthesia and all efforts were made to minimize animal discomfort. CLEAR and platinum MicroECoG arrays were implanted in male Sprague–Dawley rats ~2 months old, and male and female Thy1::ChR2/H134R-YFP (Jackson Labs 012350) and wild-type mice ~6–16 weeks old. After receiving pre-operative injections of buprenorphine (for pain management) and dexamethasone (to prevent brain swelling), animals were anaesthetized with isoflurane gas and their heads immobilized. Incisions were made over the top of the skull and craniotomies were made with a surgical drill. Electrodes were stereotactically placed on the surface of the brain, over somatosensory cortex and circular glass coverslips were applied over the top of the array, forming the cranial window. The edges of the coverslips were sealed to the skull using dental acrylic. Ground and reference wires were attached to stainless steel screws, drilled into (rats) or glued to (mice) the skull. After everything was in place, the exposed screws were covered with dental acrylic to form a smooth cephalic implant. The skin was then sutured around the implant in rats and the animals were recovered. Animals received injections of buprenorphine post surgery, as well as ampicillin antibiotic for 1 week following the implantation.

### Impedance spectra and baseline signal recordings

Following implantation of the devices, electrode site impedance spectra and baseline signal recordings were obtained. Impedance spectra were recorded at a minimum of three times per week for the duration of the implantation period in the rats. Electrode site impedance spectra were generated using the Autolab PGSTAT12 (Metrohm Eco Chemie, Utrecht, Netherlands). Baseline signal recordings were obtained using a TDT neurophysiology work system. Signals were recorded via a 32-channel active TDT headstage, plugged into the PCB connector. The headstage was connected to a TDT PZ2 amplifier, which amplified the signal before sending it to the TDT RZ2 system, from which it was sent to the computer. Three to 5 min of baseline signal data were recorded during each session. Animals were awake and behaving for the baseline signal recordings and impedance spectra collection, with the exception of experiments to test the impact of different anaesthetics on neural signal activity.

### Electrical-evoked potentials

Animals were anaesthetized with dexmedetomidine hydrochloride (0.05 mg kg^−1^, Orion Pharma) and their hindlimbs shaved. Two adhesive surface electrodes were attached to one leg at a time and held in place with tape. These electrodes were hooked up to a stimulation box (A-M Systems Isolated Pulse Stimulator, Model 2100) linked to the TDT RZ2 system via a BNC (Bayonet Neill-Concelman) cable. The animal’s electrodes were plugged into the RZ2 system via the TDT headstage and PZ2 amplifier. In this way, an electrical stimulus was sent to the animal’s sciatic nerve from the RZ2 system, and the response in the somatosensory cortex was recorded through the CLEAR device and sent back to the computer. After completion, the animals were recovered with an injection of atipamezole hydrochloride (0.3 mg kg^−1^, Orion Pharma).

### Optogenetic testing

CLEAR and platinum micro-ECoG arrays were implanted on the cortex of Thy1::ChR2 mouse (Jackson Labs, 012350), but in this case no window was placed over the array. Instead, the brain was left open and an optical fibre attached to a laser (Laserglow Technologies, Ontario, Canada) was brought into close proximity with the cortex (Fig. 4a). The anaesthesia was switched from isoflurane, which inhibits neural signalling, to a combination of ketamine (75 mg kg^−1^) and dexmedetomidine (25 μg kg^−1^). High-intensity blue light with a maximum power of 63.7 mW mm^−2^ was then directed onto various regions of the brain through the CLEAR device (Fig. 4b), while simultaneously recording the neural response to the optical stimulation. Once experimentation was complete, the animals were killed with an intraperitoneal injection of Fatal PLUS pentobarbitol solution and a control experiment was conducted.

### *In vivo* imaging

Imaging took place on a Leica MZ 16F stereoscope. Animals were anaesthetized with a combination of isoflurane gas and dexmedetomidine hydrochloride (0.05 mg kg^−1^, Orion Pharma), and kept on a heated water blanket. The animals’ heads were stabilized to prevent breathing artefacts. Animals were injected with 12 mg ml^−1^ fluorescein isothiocyanate-labelled dextran dissolved in PBS to make the blood vessels fluorescent under blue light. Bright-field and fluorescent images were taken of the electrode arrays and surrounding brain tissue. In addition, blood flow movie recordings were acquired using the light path of the Leica MZ 16F stereoscope in combination with the Sony HDR-SR11 high-definition camcorder.

### Optical coherence tomography

A custom spectral domain OCT system was used to obtain 3D OCT angiograms and velocity profiles. The spectral domain OCT system used a light source at the central wavelength of 1,300 nm and spectral bandwidth of 200 nm, delivering 10 mW of optical power at the tip of a single-mode fibre. The system provides 5 μm axial and 4 μm lateral resolution with a × 10 telecentric lens.

The cross-sectional OCT angiograms were obtained by recording ten OCT B-scans at each cross-section and employing a phase-sensitive angiography technique to the OCT data. For the field of view of 2.8 × 2.8 mm^2^, 650 cross-sectional angiograms were obtained while each one consisted of 650 lateral positions. For the field of view of 1.1 × 1.1 mm^2^, the number of cross-sections and the number of lateral positions were 500. After stacking all cross-sectional angiograms to form the volume angiogram, a 3D blurring kernel was applied to reduce the noise and then the two-dimensional maximum intensity projection of the angiograms was obtained. Adaptive histogram equalization (MATLAB and Image processing toolbox Release 2012b, The MathWorks, Inc., Natick, Massachusetts, United States) was applied to improve the contrast of maximum intensity projection images. For 3D visualization purposes, contrast enhancement and vessel de-shadowing was performed by applying a biased sigmoid nonlinear transform to the intensity of 3D angiography data. The bias of the sigmoid function was defined to value at which this function has a value of 0.5. At each point, the bias of the sigmoid function was determined according to the angiogram values above it.

For blood velocity measurement, the tissue was scanned at 3,500 (Field of View (FOV)=2.8 × 2.8 mm^2^) or 2,000 (FOV=1.1 × 1.1 mm^2^) A-scans per cross-section. The scans were performed at 500 tissue cross-sections and rate of 40,000 A-scans per second. At each position, the Doppler shift introduced by the moving particles was estimated by calculating the average power spectrum density of a signal consisting of seven consecutive OCT signals at that position (*ω*_a_). Axial velocity at that depth was then calculated by 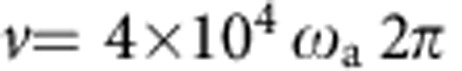.

## Author contributions

D.-W.P., A.A.S., S.M., S.K.B., T.J.R., J.P.N., F.A., S.T.F., R.P., S.T., Z.M. and J.C.W. performed the research. D.-W.P., A.A.S., S.K.B, F.A., Z.M. and J.C.W. wrote the manuscript. Z.M. and J.C.W. designed the research.

## Additional information

**How to cite this article:** Park, D.-W. *et al.* Graphene-based carbon-layered electrode array technology for neural imaging and optogenetic applications. *Nat. Commun.* 5:5258 doi: 10.1038/ncomms6258 (2014).

## Supplementary Material

Supplementary Figures and References Supplementary Figures 1-10 and Supplementary References

Supplementary Movie 1 Blood flow movie recordings through the graphene electrode sites in different rats.

Supplementary Movie 2 Blood flow movie recordings through the graphene electrode sites in different rats.

Supplementary Movie 3 3D optical coherence tomography (OCT) image for the CLEAR device.

Supplementary Movie 4 3D optical coherence tomography (OCT) image for the platinum device.

## Figures and Tables

**Table 1:** Number of *in vivo* experiments for each purpose

**Implant description**	***In vivo*** **vascular imaging**	**Impedance spectroscopy**	**Baseline signal recordings**	**Electrical evoked potentials**	**Optogenetic experiments**
Rat CLEAR device (chronic)	3	4	4	4	—
Rat Pt micro-ECoG device (chronic)	3	3	3	3	—
Mouse CLEAR device (terminal)	3	—	3	—	3
Mouse Pt micro-ECoG device (terminal)	2	—	2	—	2

CLEAR, Carbon Layered Electrode Array; ECoG, electrocorticography.

Table 1 describes the number of devices that were implanted during this study and the experiments they were used for. Multiple rats were implanted bilaterally, one device on each brain hemisphere, to serve as better within animal comparisons and to reduce total animals numbers used. Three rats were implanted bilaterally with one CLEAR micro-ECoG device and one platinum micro-ECoG device and one rat was implanted with a single CLEAR device. All rats were chronic studies. Five mice were implanted during terminal procedures. Three animals had CLEAR and platinum micro-ECoG devices implanted, and two had only the CLEAR device.
