# Supplementary material for: Graphene-based carbon-layered electrode array technology for neural imaging and optogenetic applications
Source: Nat Commun. 2014 Oct 20;5:5258. doi: 10.1038/ncomms6258 (PMC4218963; doi:10.1038/ncomms6258)
Supplement: Supplementary Figures and References — Supplementary Figures 1-10 and Supplementary References [file ncomms6258-s1.pdf]

Supplementary figure 1| Graphene Growth and Transfer

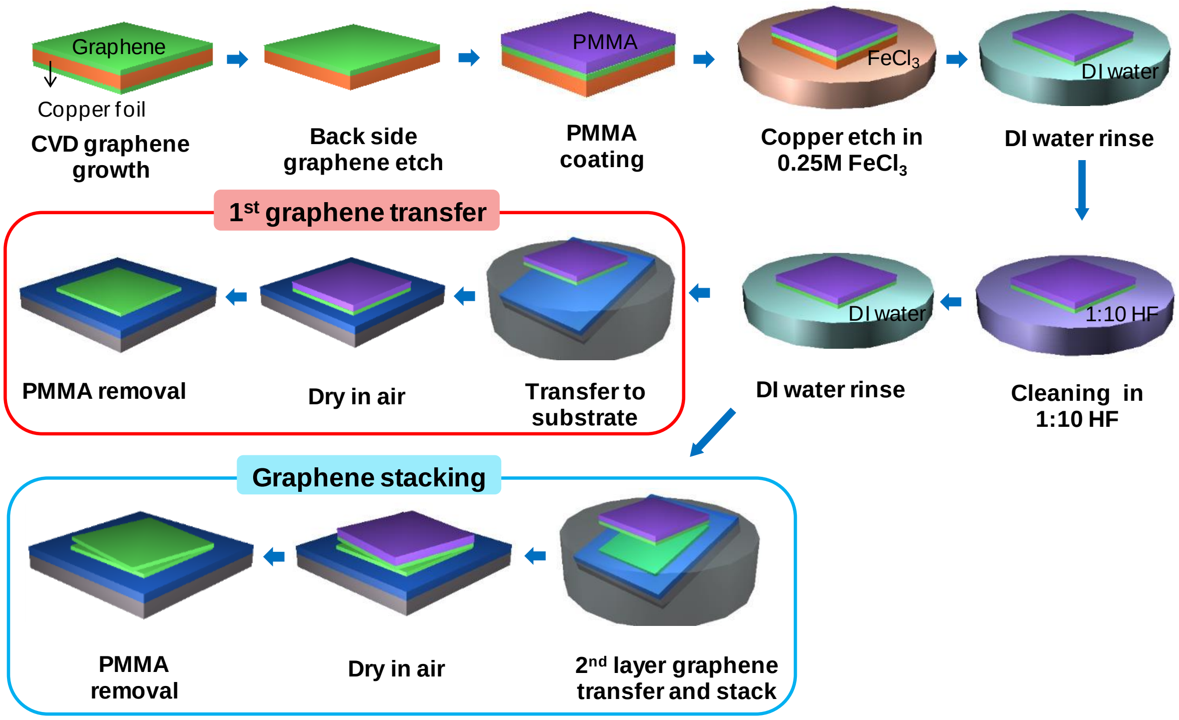

Figure 1: Graphene release, transfer and stacking processes. The graphene stacking began with CVD graphene growth on a copper (Cu) foil. Mono-layer graphene was grown on both the top and bottom sides of the Cu foil. To utilize the top side graphene, back side graphene was etched using oxygen (O2) plasma (50W, 10 sccm O_2, 10 mTorr, for 1 minute). The top side graphene was coated by 950k PMMA (polymethyl methacrylate) C4 (MicroChem) to protect the one-atom thick graphene layer. The Cu was etched in 0.25 M ferric chloride ($FeCl_3$) for 3 hours and rinsed in de-ionized (DI) water. The graphene was cleaned in 1:10 hydrofluoric (HF) acid for 1 hour to remove the copper composite residues and rinsed in DI water. The first graphene transfer was then performed on the desired substrate. After drying the PMMA/graphene sample in a N_2 atmosphere dry box, the PMMA was removed using acetone and the sample was rinsed in DI water. Following the above procedure, the graphene stacking was performed on the previously graphene-transferred substrate. Multiple layers of graphene could be stacked on the desired substrate using this method.

Supplementary figure 2| Images of CVD Graphene

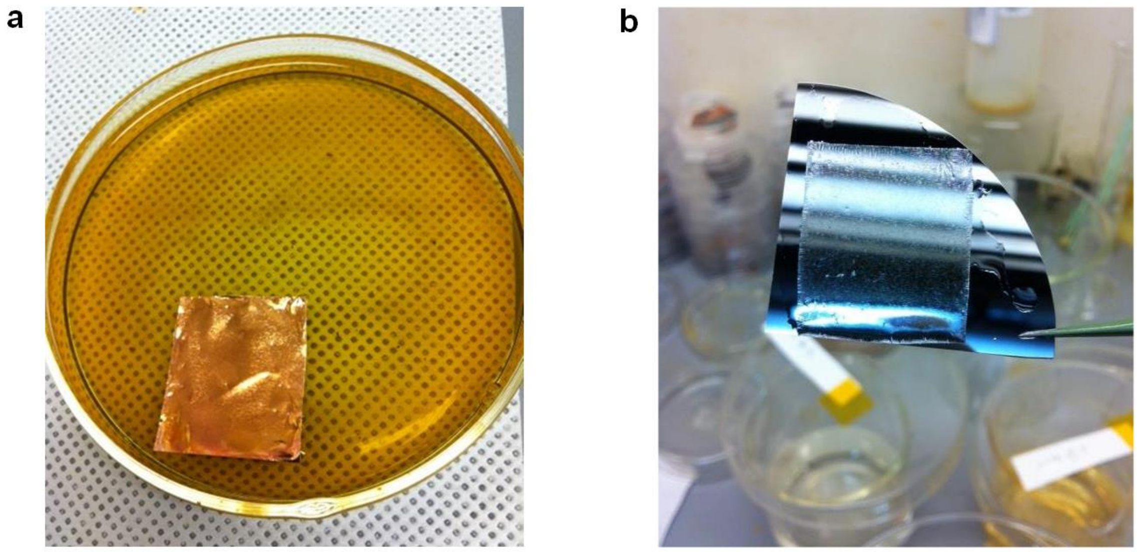

Figure 2. CVD Graphene Images. The CVD graphene was grown on a 2 cm x 3 cm piece of copper (Cu) foil. **a.** PMMA/graphene/Cu on Cu etchant ($FeCl_3$). **b.** After the Cu was completely etched, the PMMA/graphene sheet was moved to DI water using a piece of silicon wafer.

Supplementary figure 3| Wetting property control of Parylene C

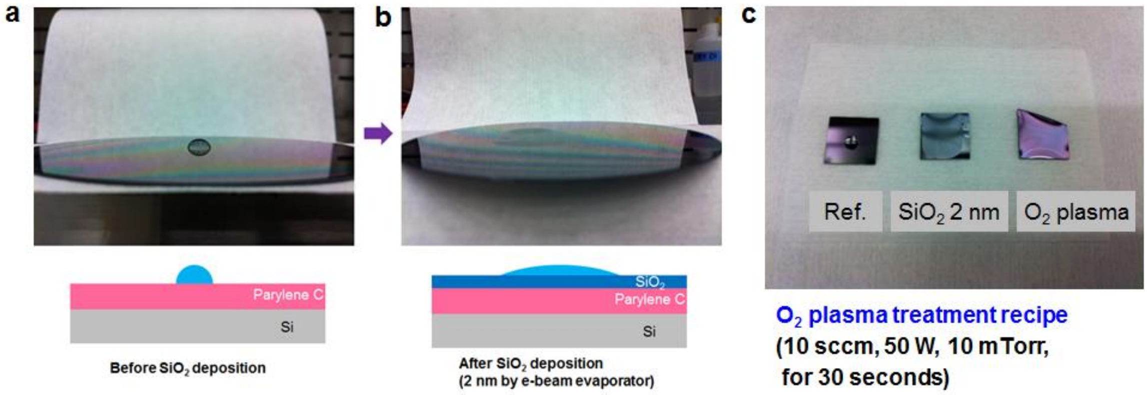

Figure 3. Wetting property changes of Parylene C after oxygen plasma treatment and SiO_2 deposition. The Parylene C surface was changed from hydrophobic to hydrophilic for favorable graphene transfer. **a.** Water droplet on hydrophobic Parylene C substrate prior to SiO_2 deposition. **b.** Water droplet on hydrophilic Parylene C substrate post SiO_2 deposition. **c.** Comparison of wetting properties for bare Parylene C, Parylene C with 2 nm of e-beam evaporated SiO_2, and oxygen plasma-treated (10 sccm, 50 W, 10 mTorr, 30 sec) Parylene C[52]. Plasma treatment was used in this study.

Supplementary figure 4| Graphene sheet transfer onto Parylene C coated wafer

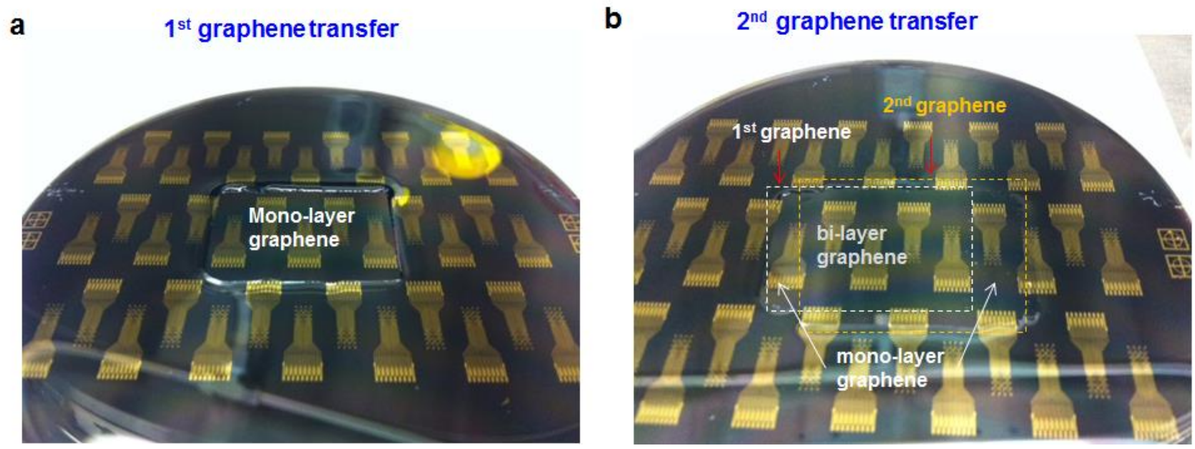

Figure 4. Graphene sheets after transfer onto pre-processed wafer surfaces. **a.** Monolayer graphene sheet after transfer onto a pre-processed Parylene-coated silicon wafer. DI water was poured onto the surface of the wafer for verification of the existence of the graphene sheet. Since graphene is intrinsically hydrophobic, due to the coherent bonding of carbon atoms, water does not spread onto the sheet, but instead flows onto the surrounding hydrophilic, plasma treated, Parylene C. **b.** The same wafer shown in S4a after transfer of a second graphene sheet. The second graphene sheet was intentionally misaligned in this case to make both monolayer and bilayer regions, for comparison tests.

Supplementary figure 5| Detailed fabrication process

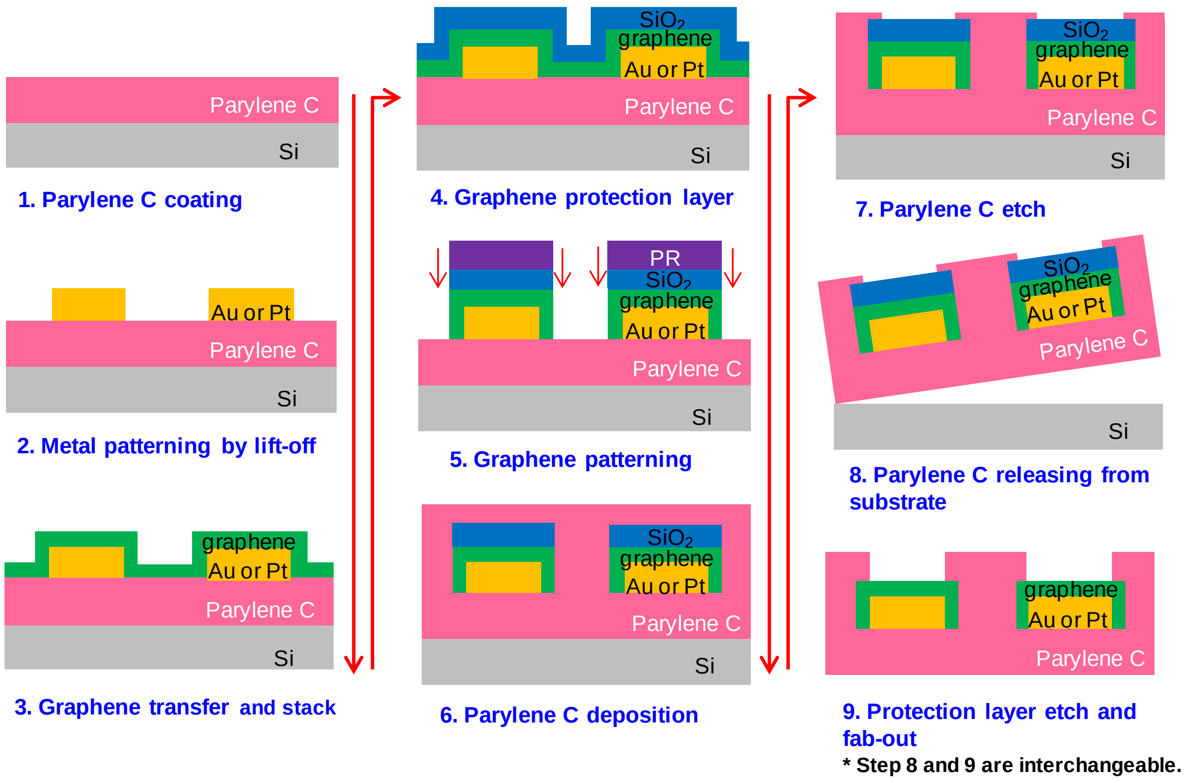

Figure 5: Detailed fabrication process flow illustration. Parylene C (15 μm) was coated on a 4-inch silicon (Si) wafer using Parylene deposition equipment (PDS 2010 Labcoter 2, Specialty Coating Systems Inc.). Electrode sites, traces, and pads were using Shipley 1813 photoresist and lift-off techniques. Gold (Au) metal was used with a chromium (Cr) adhesion layer. For the CLEAR device, only the pads and parts of the traces were patterned with metal, leaving the area of the device which would be in contact with the brain to be patterned with transparent graphene electrode sites and traces. Before graphene transfer, oxygen (O_2) plasma treatment was performed to transform the Parylene C surface from hydrophobic to hydrophilic. Graphene was then transferred to the pre-patterned substrate using a wet transfer technique. To protect the graphene from possible damage during subsequent process steps, 30 nm SiO_2 was deposited using e-beam evaporation. Then the SiO_2/graphene layers were patterned using photolithography and dry etching. For the SiO_2 etch, reactive-ion etching (RIE) (Unaxis 790 dry etcher) was used with CF_4 gas at 45 sccm and O_2 gas at 5 sccm, 100W power, and 40mTorr pressure for 90 seconds. For the graphene etch, RIE with O_2 gas at 10 sccm, 50W power, and 10mTorr pressure was used for 60 seconds. After the photoresist was stripped, a second Parylene C

layer (10 µm) was evaporated for encapsulation. The Parylene C was then patterned using two RIE steps with O_2 plasma. The first RIE step etched the device outline and the second RIE step etched outline, electrode sites, and pads. Next, the wafer was soaked to release the devices from the silicon substrate. Finally, the SiO_2 protection layer was etched using 1:6 buffered oxide etchant (BOE) for 60 seconds, and the devices were rinsed thoroughly with DI water.

Supplementary figure 6| Graphene Characterization using Raman spectroscopy

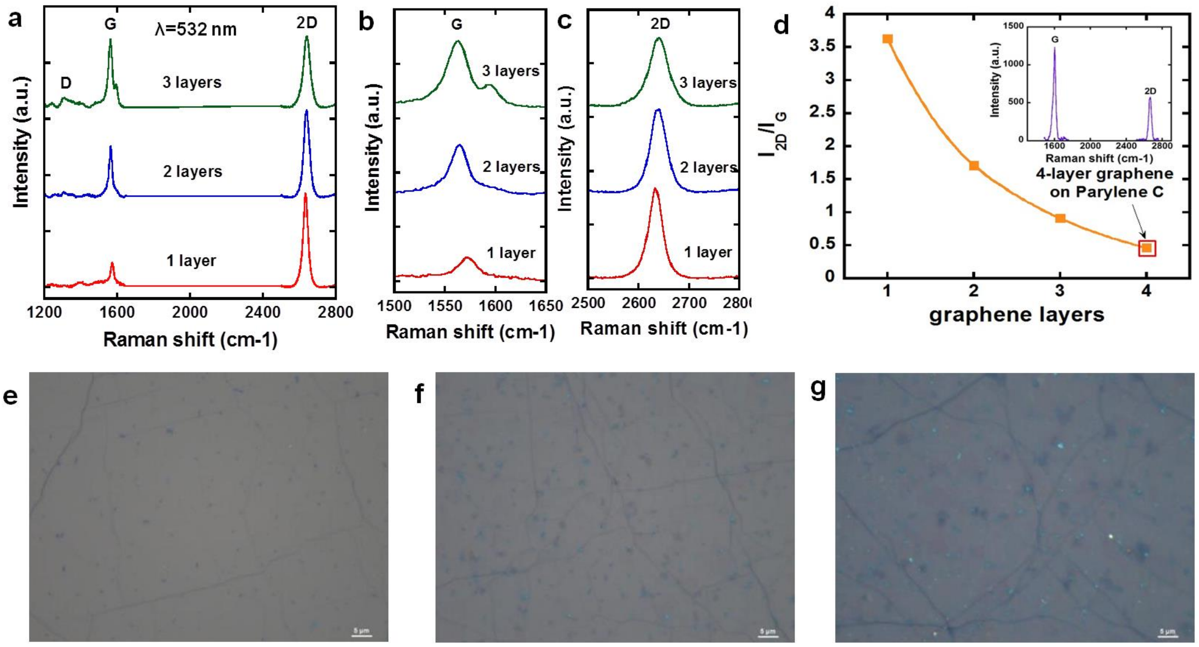

Figure 6. Raman spectroscopy of stacked graphene. **a.** G and 2D peaks for 1, 2, and 3-layer graphene. **b** and **c.** Enlarged G and 2D peak regions, respectively. **d.** G and 2D peak ratio (I_{2D}/I_G) trend versus number of graphene layers. The Raman spectroscopy was carried out with a Horiba LabRAM ARAMIS Raman system using a 532 nm green laser and a 100x objective. Microscope images of **e.** 1 layer, **f.** 2 layers, and **g.** 3 layers of graphene.

The mono-layer graphene and multi-layer graphene samples were characterized using Raman

spectroscopy and current-voltage (I-V) measurement on a SiO_2/Si substrate. Raman spectroscopy has been used as a fundamental method to investigate phonon characteristics of graphene, and can aid in identifying the existence of graphene on a particular substrate and the number of graphene layers present[54,55]. The intensity ratio change of the 2D and G peaks for 1, 2, and 3-layer graphene verified the viability of the graphene stacking process, showing the unique phonon characteristics of graphene layers (Figure S6). The single sharp G peak is characteristic of the two-dimensional sp^2 bonded carbon atoms of graphene. For the 1 layer graphene, the G peak was located at 1572.5 cm^{-1}, and for the 3-layer graphene, the G peak was at 1563.3 cm^{-1}. Therefore, as the number of graphene layers increased, the G peak showed a left shift (redshift). The 2D peaks were at 2633 cm^{-1} for the 1 layer graphene and at 2640.7 cm^{-1} for 3-layer graphene, showing that the 2D peak experienced a right shift (blueshift) with increasing graphene layers. These peak shift trends are consistent with the previous Raman study for multilayer graphene[54,55].

The 2D to G peak ratio (I_{2D}/I_G) trend shown in Figure S6(d) demonstrates the changes occurring with the addition of each graphene layer. For the 1 layer graphene, the G peak intensity was the lowest, while the 2D peak intensity was the highest with $I_{2D}/I_G = 3.63$. As the number of graphene layer was increased, the G peak intensity was increased and the 2D peak intensity was decreased, resulting in I_{2D}/I_G ratios for 2-layer and 3-layer graphene were 1.43 and 0.91, respectively. This decreasing I_{2D}/I_G ratio is characteristic of multilayer graphene samples[1,2]. It is also important to note that the D peak near 1350 cm^{-1}, which is generally a result of sample defects, was not significant for the graphene layers[54,55]. This proves that the CVD graphene grown for this study did not have a significant number of defects before and after stacking.

Supplementary figure 7| Graphene sheet resistance measurement

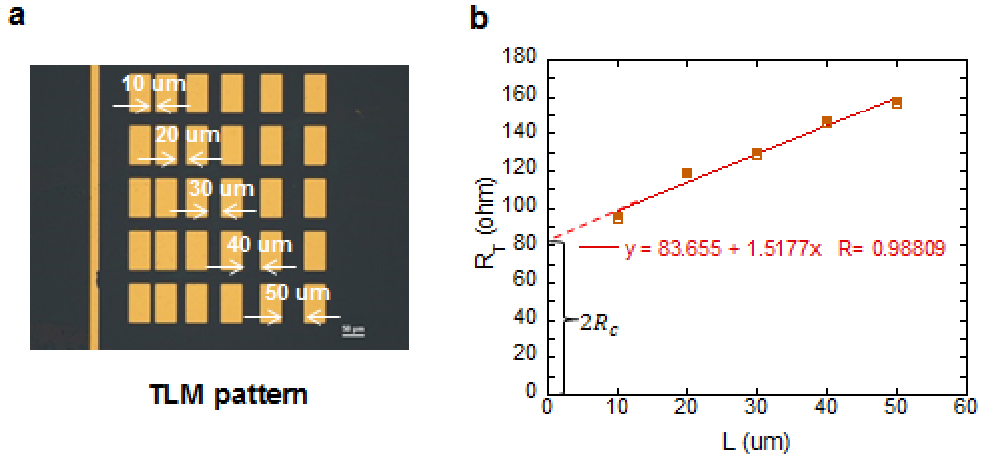

Figure 7. Transmission line method (TLM) for sheet resistance extraction. **a.** Fabricated TLM pattern with graphene channel length from 10 to 50 μm, the channel width is 100 μm. **b.** Total resistance versus channel length for monolayer graphene.

To measure the sheet resistance of the graphene, transmission line method (TLM) was performed[56]. Figure S7a shows the fabricated TLM pattern with graphene channel lengths from 10 μm to 50 μm. For etch length, five samples were measured and the average values are plotted. Figure S7b shows the total resistance (R_T) versus the length (L) plot for monolayer graphene. Using the TLM theory, the sheet resistance can be extracted from the following equation:

$$R_T = \frac{R_s}{W} \cdot L + 2R_c = slope \cdot L + 2R_c$$

Where R_T is the measured total resistance, R_S is the sheet resistance, R_C is the contact resistance, W and L are the width and length of the TLM pattern, respectively.

From the equation, the slope of the graph is as follows:

$$slope = \frac{R_s}{W}$$

Now, the sheet resistance can be extracted from the slope of the linear fit line as below:

$$R_s = slope \cdot W = 1.5177 \times 100 = 151.77\ \Omega/\square$$

Similarly, sheet resistance of the stacked graphene can be extracted using the TLM.

Supplementary figure 8| Additional baseline signal and evoked potential results

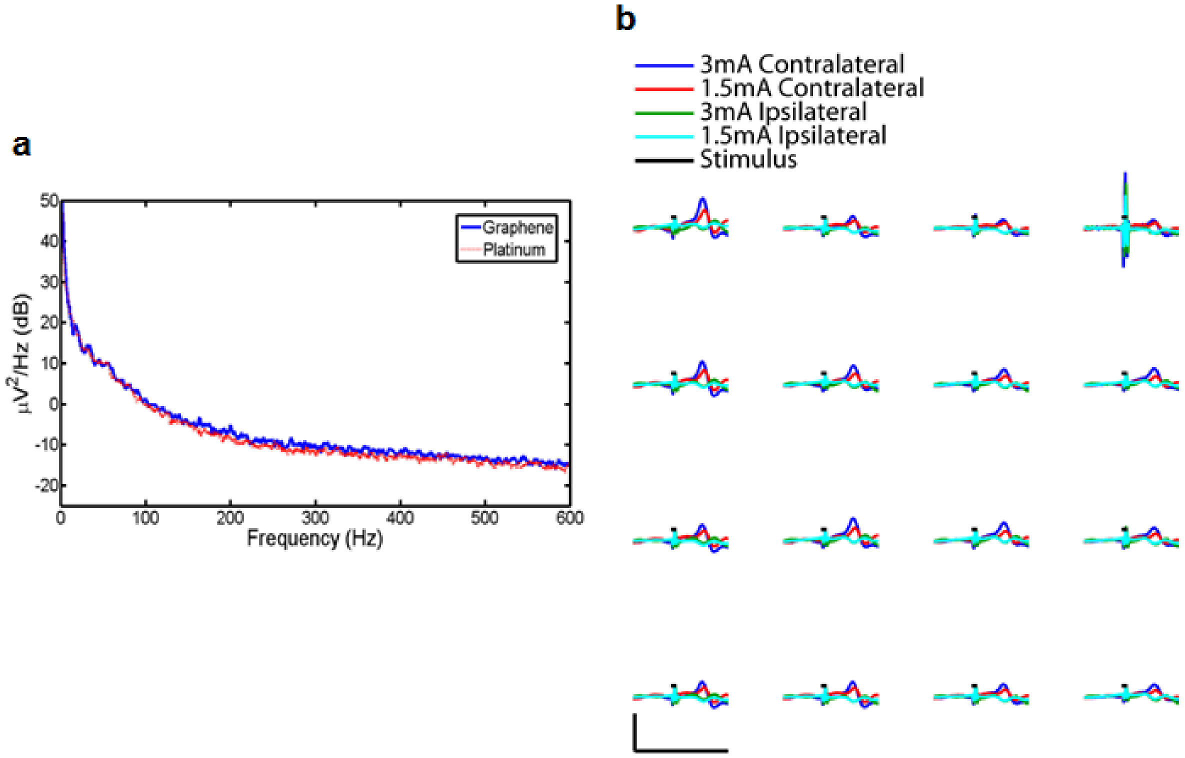

Figure 8. a. Power spectra for the baseline signals recorded by single channels on the CLEAR and platinum micro-ECoG devices in the 1-600 Hz frequency range (*in vivo*). **b.** Sensory evoked potentials recorded by a CLEAR device, via electrical stimulation of the sciatic nerve on the hind leg of the rat, both contralateral and ipsilateral to the array. The device was implanted over somatosensory cortex. Stimuli were applied for 1 ms at 3 mA and 1.5 mA current levels. X-scale bar represents 50 ms, y-scale bar represents 100 uV.

Supplementary figure 9| Additional optogenetic experiment results

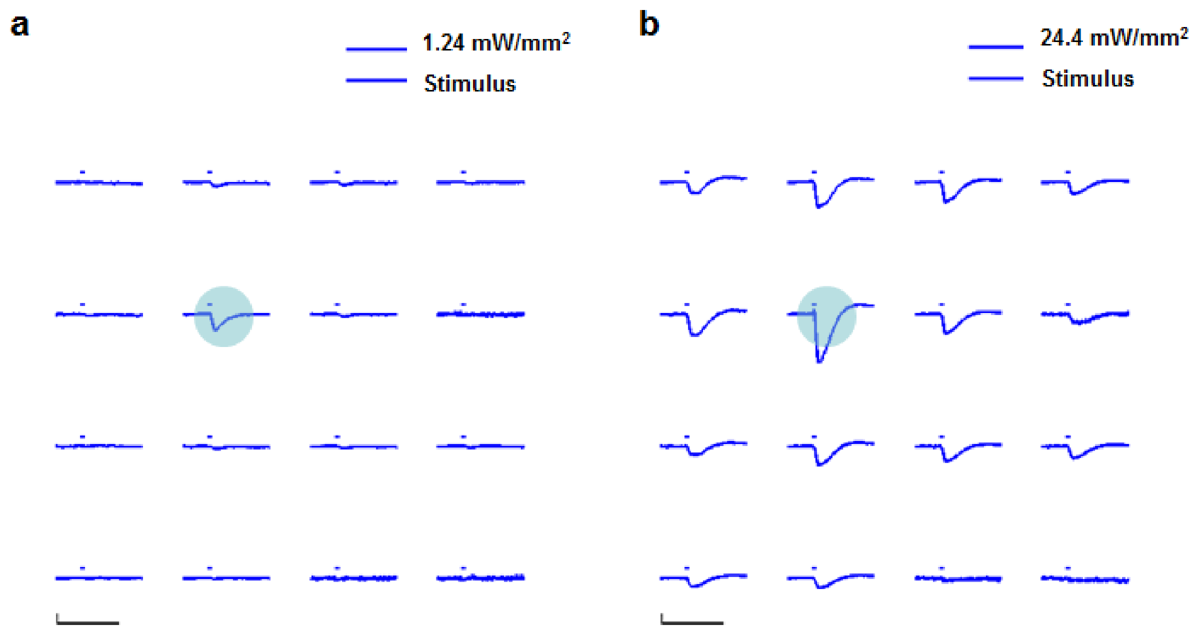

Figure 9. a. Optogenetic experiment using a blue laser at 473 nm wavelength with 1.24 mW/mm^2 intensity on a CLEAR device. **b.** Optogenetic experiment using the same laser as figure S8c but with a change of intensity to 24.4 mW/mm^2 on a CLEAR device.

Supplementary figure 10| Additional artifact experiment results

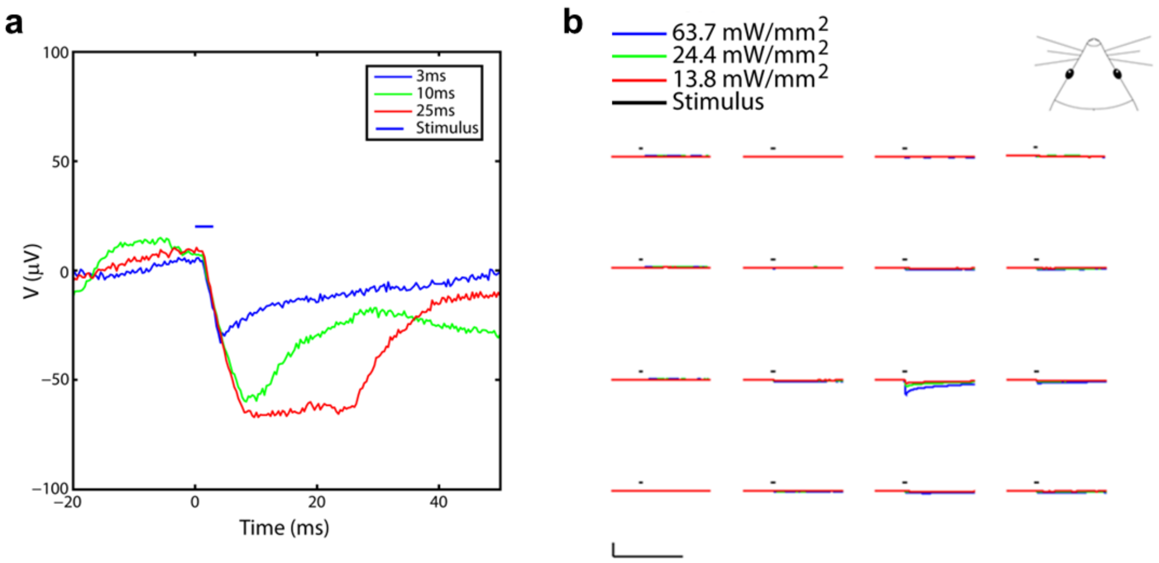

Figure 10. a. Artifact experimental results dependent on light stimulus time duration varying from 3 ms to 25 ms tested in a wild type chronically implanted rat with graphene electrode site. **b.** Artifact experimental results dependent on the light stimulus power ranging from 13.8 mW/mm^2 to 63.7 mW/mm^2 done in cadaver ChR2 mouse.

References

53. Song, J. S., Lee, S., Jung, S. H., Cha, G. C. & Mun. M. S. Improved Biocompatibility of Parylene-C Films Prepared by Chemical Vapor Deposition and the Subsequent Plasma Treatment. *Journal of Applied Polymer Science*. **112**, 3677-3685 (2009)
54. Ferrari, A. C. *et al.* Raman Spectrum of Graphene and Graphene Layers. *Phys. Rev. Lett.* **97,** 187401 (2006).
55. Ferrari, A. C. Raman spectroscopy of graphene and graphite: Disorder, electron–phonon coupling, doping and nonadiabatic effects. *Solid State Commun.* **143,** 47–57 (2007).
56. Schroder, Dieter. Semiconductor material and device characterization, 3rd edition, chapter **3**, 138-149, John Wiley (1998).
